# Undergraduate Medical Competencies in Digital Health and Curricular Module Development: Mixed Methods Study

**DOI:** 10.2196/22161

**Published:** 2020-10-29

**Authors:** Akira-Sebastian Poncette, Daniel Leon Glauert, Lina Mosch, Katarina Braune, Felix Balzer, David Alexander Back

**Affiliations:** 1 Department of Anesthesiology and Intensive Care Medicine Charité – Universitätsmedizin Berlin Corporate member of Freie Universität Berlin, Humboldt-Universität zu Berlin, and Berlin Institute of Health Berlin Germany; 2 Einstein Center Digital Future Berlin Germany; 3 Department of Paediatric Endocrinology and Diabetes Charité – Universitätsmedizin Berlin Corporate member of Freie Universität Berlin, Humboldt-Universität zu Berlin, and Berlin Institute of Health Berlin Germany; 4 Department of Traumatology and Orthopaedics, Septic and Reconstructive Surgery Bundeswehr Hospital Berlin Berlin Germany; 5 Dieter Scheffner Center for Medical Education and Educational Research Charité – Universitätsmedizin Berlin Corporate member of Freie Universität Berlin, Humboldt-Universität zu Berlin, and Berlin Institute of Health Berlin Germany

**Keywords:** digital health, eHealth, mHealth, digital health education, elective module, eHealth education, curriculum, medical school, digital health mindset, qualitative research, interview, survey

## Abstract

**Background:**

Owing to an increase in digital technologies in health care, recently leveraged by the COVID-19 pandemic, physicians are required to use these technologies appropriately and to be familiar with their implications on patient care, the health system, and society. Therefore, medical students should be confronted with digital health during their medical education. However, corresponding teaching formats and concepts are still largely lacking in the medical curricula.

**Objective:**

This study aims to introduce digital health as a curricular module at a German medical school and to identify undergraduate medical competencies in digital health and their suitable teaching methods.

**Methods:**

We developed a 3-week curricular module on digital health for third-year medical students at a large German medical school, taking place for the first time in January 2020. Semistructured interviews with 5 digital health experts were recorded, transcribed, and analyzed using an abductive approach. We obtained feedback from the participating students and lecturers of the module through a 17-item survey questionnaire.

**Results:**

The module received overall positive feedback from both students and lecturers who expressed the need for further digital health education and stated that the field is very important for clinical care and is underrepresented in the current medical curriculum. We extracted a detailed overview of digital health competencies, skills, and knowledge to teach the students from the expert interviews. They also contained suggestions for teaching methods and statements supporting the urgency of the implementation of digital health education in the mandatory curriculum.

**Conclusions:**

An elective class seems to be a suitable format for the timely introduction of digital health education. However, a longitudinal implementation in the mandatory curriculum should be the goal. Beyond training future physicians in digital skills and teaching them digital health’s ethical, legal, and social implications, the experience-based development of a critical digital health mindset with openness to innovation and the ability to assess ever-changing health technologies through a broad transdisciplinary approach to translate research into clinical routine seem more important. Therefore, the teaching of digital health should be as practice-based as possible and involve the educational cooperation of different institutions and academic disciplines.

## Introduction

### Background

With the progress in the introduction of digital solutions in patient care, such as electronic health records [1-3], artificial intelligence (AI) for decision support [4,5], telemedicine [6-8], or robotic surgery [9], the need for physicians and other health care professionals to get familiar with digital health is increasing. The recent COVID‑19 pandemic has highlighted the advantages of remote care and puts pressure on health care professionals and infrastructure to adapt to a fast-developing, globalized world [10].

Although the introduction of new technologies in medicine is accompanied by public hope for better and more efficient patient care [11,12], experts agree that a new technology can only be as good as the physician using it [13]. Implementing digital health technologies into clinical settings remains a prolonged process [14], with one of the major barriers being the lack of health professionals’ knowledge and awareness of the new technologies and the skills to use them [15].

Training physicians and nurses in the practical use of digital technologies at an early stage of their career is overdue to prepare them for their future challenges [3,13,16]. In this context, data literacy is considered a decisive skill for health care workers [17]. Digital literacy of students and young physicians, who are often referred to as the generation of digital natives, is discussed ambivalently in the literature. Although some authors postulate that growing up with digital services may lead to differentiated use in a professional context [18], other publications stress that the implication of the digital native stereotype would leave learners unsupported and technologies used in inappropriate ways, making further research in this area indispensable [19].

The relevance of implementing digital health education in medical curricula is evident [16]. Various pilot projects have been described in the literature, with differences in length and focus [20,21]. All of them reported a high level of satisfaction among students attending digital health–related courses, with expected positive influences on their skillset in later professional life [22].

According to a Europe-wide survey, medical students felt lacking digital literacy and demanded a wider implementation of digital health topics in their curricula [23,24]. Along with the need for education on ethics and technology specifics, a general introduction to the topic and the teaching of basic aspects of the field were asked for [23,24]. In 2019, the deans of 25 European medical universities agreed on the rapid implementation of digital health education in their respective medical schools’ curricula, focusing on interprofessional education, practical skills, and innovation [25]. To scale up the implementation of digital health in health care education, there is still a need to provide and exchange best practices of digital health in medical education. The ongoing public funding of digital health research projects has so far not been correlated with an increase in the number of corresponding courses in the medical curriculum [23]. The implementation of teaching initiatives for digital competencies should urgently be improved.

### Aim

We intended to deliver a proof of concept of teaching digital health at a medical school, describing the development, introduction, and evaluation of a 3-week elective module. We further aimed to identify undergraduate medical competencies in digital health and their suitable teaching methods.

## Methods

### Ethics Approval and Consent to Participate

Ethical approval for this study was granted by the ethics committee of the Charité—Universitätsmedizin Berlin (EA1/236/19). Participation in the study was voluntary. Before the study, all participants provided their consent.

### Study Setting

This mixed methods study took place at the medical school of a large German university hospital, the Charité—Universitätsmedizin Berlin, in the context of the development and deployment of a pioneer teaching module for digital health, which was realized in January 2020. At the time of the study, approximately 7500 students were enrolled at the medical school [26]. Although medical informatics was taught as an independent study program since 1972 in Heidelberg, Germany, and was mentioned in the Medical Licensure Act (*Approbationsordnung für Ärzte*) since 1989, medical informatics is only marginally taught in the German medical curriculum. The terms *digital* or *digital health* are still not mentioned in the Medical Licensure Act in Germany. By the end of the third year of the undergraduate modular medical track, students at the Charité have to choose an elective module from a variety of 3-week course programs covering different medical fields that are usually not covered by the mandatory curriculum. The modules are in direct competition for the students’ interest and comprise 60 teaching hours (45 min each). Only the modules selected the most often by the students take place.

Over the course of 2 years, we developed a teaching module for digital health based on qualitative results of semistructured interviews with experts described below and by reviewing similar existing projects. The module was designed to change over time, flexibly reacting to the feedback received and to new scientific findings. In multiple face-to-face meetings, calls, and emails with potentially interested parties, we identified 32 lecturers from 16 departments of our university hospital as well as from 6 academic and nonacademic partner organizations who were willing to participate.

The cooperation with the lecturers led to a high variety of digital health topics, such as lessons on law, ethics, and economics as well as on digital pharmacology or AI and big data in research. In addition to the theoretical teaching in seminars, our main focus was to provide hands-on experience with digital health technologies. Practice units on smart implants and wearables, symptom checker apps, telecardiology, mixed reality-assisted surgery, and video consultations were included with the help of experts and developers of the corresponding technologies. To enable the students to play the roles of different health system stakeholders, we encouraged them to compete in groups during a mini-hackathon. During the course of the whole module, each student group invented and eventually pitched a new product for a specific problem in health care. Further details and lessons can be found in the module timetable (Multimedia Appendix 1).

### Study Design

We chose a mixed methods approach consisting of an abductive, qualitative study based on semistructured interviews and a cross-sectional survey study using a web-based questionnaire. Qualitative data included the interview transcripts and results from the open-ended questions of the questionnaire. Items from the questionnaire with a five-point Likert-type scale as a response format were considered quantitative data.

### Data Collection

Between October and December 2019, DG conducted semistructured interviews with 5 experts in digital health and medical education. Purposive sampling was deployed on the research team’s professional external networks to select experts with complementary backgrounds in Europe.

We developed the interview guideline according to the research question (Textbox 1) and tested and adjusted the questions during pilot interviews within the research team. The interviews were conducted in English or German via phone calls and were recorded and transcribed verbatim by the interviewer (DG). Interview transcripts were reviewed by AP and LM. The median interview length was 31 min (range 27-50 min).

Furthermore, we collected feedback on the module and further digital health topics from the participating lecturers and via corresponding surveys. Survey items were generated through a literature review and informal research meetings (Multimedia Appendix 2). We grouped 17 items into 3 topics and chose a five-point Likert-type scale as an ordinal response format, with the options strongly agree (1), agree (2), neutral (3), disagree (4), and strongly disagree (5), and 4 open-ended questions. Respondents were also given space to comment on each topic. The 3 topics were (1) digital health at medical schools, (2) experience from the digital health module, and (3) feedback on the organization of the digital health module.

Pretests within the research team did not alter the questionnaire. Through pilot tests with associated research colleagues, clarity, relevance, and arrangement of the questionnaire items were improved.

The survey questionnaire was given to the participated students on the last day of the module as a paper version and slightly modified for participating lecturers of the module. For the latter, we sent an email invitation with multiple reminders to participate in the survey to all 32 lecturers. This web-based survey data were collected and managed using REDCap (Research Electronic Data Capture) tools hosted at Charité—Universitätsmedizin Berlin [27,28]. No incentive or compensation was given to the survey participants.

**Guideline questions for the semistructured interviews.**

1. What is your relation to digital health and what are you currently dedicating to professionally?
2. How do you think will digitalization affect doctors' work in the future, and what are the biggest challenges?
3. What topics of digital health would you recommend to teach at German medical schools?
4. Which teaching methods would you imagine or consider to be the most effective for teaching digital health?
5. How can awareness for the challenges of digital health be maximized among medical students?
6. Based on your own experience, do you have further advice on what factors should be considered when teaching digital health?

### Data Analysis

We performed an abductive analysis of the interview transcripts and the results of the open-ended questions of the questionnaire from the lecturers and students to identify the predominant themes [26]. Predefined themes included teaching format and learning objectives. The theme consisted of multiple codes that were later defined as subthemes. The saturation of codes was achieved with a growing number of interviews to ensure covering the majority of the aspects connected to our research question. All coding was performed and reviewed by DG, AP, and LM using MaxQDA qualitative data analysis software (MaxQDA 18.3.2; VERBI GmbH).

Descriptive data analysis of questionnaire items was conducted using Microsoft Excel 2020 (version 16.35).

## Results

### Overview

**Figure 1:**
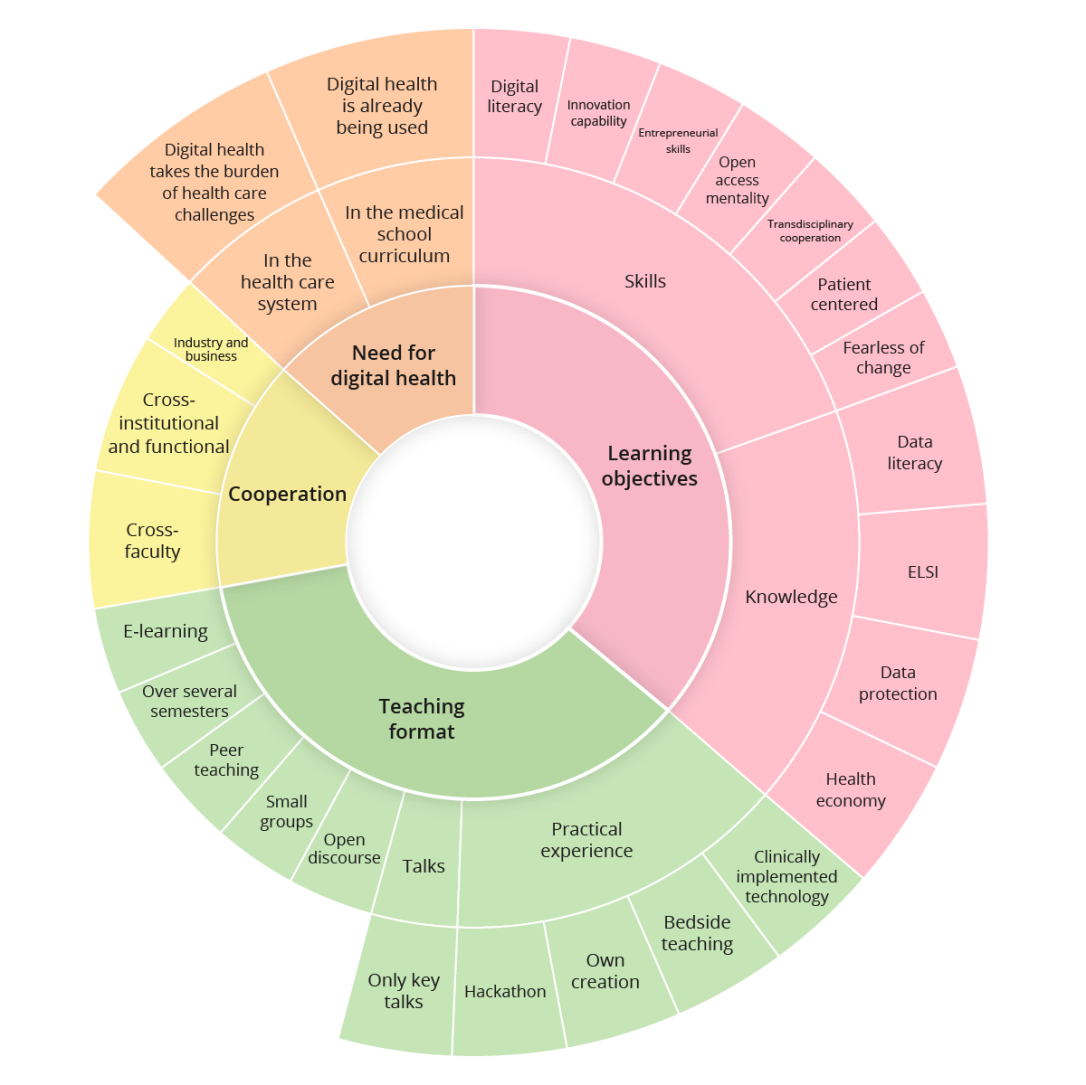
This sunburst diagram represents the qualitative results. Within the 4 themes (inner ring), subthemes (middle ring) are assigned and specified (outer ring). ELSI: ethical, legal, and social implications.

We constructed this mixed methods study based on 5 interviews with digital health experts and a questionnaire involving lecturers and students. The sunburst diagram (Figure 1) visualizes the qualitative results, specifically the 4 themes in the inner ring with the most relevant subthemes in the middle ring that are specified in the outer ring.

The quantitative data were represented by the questionnaires, where the response rate was 91% (10/11) for the students and 100% (32/32) for the lecturers.

### Qualitative Results

#### Need for Digital Health

##### In the Health Care System

According to the respondents, the positive effects of digital health on the health care system were clearly visible in areas such as communication, documentation, and patient empowerment. Digital health technologies would be already integrated into physicians’ everyday lives and were indispensable, as younger patients, in particular, would explicitly demand for them.

##### In the Medical School Curriculum

It was stated that the health care system of the future would be highly digitalized. It would, therefore, be necessary to prepare medical students accordingly as early as possible. Digital health would already be part of today’s clinical practice, and concerns were expressed that a lack of knowledge in this field would lead to individual failure. Medical students would have to lose their initial reservations and become critical experts of digital health as much as their analog counterparts.

#### Learning Objectives—Skills

##### Digital Literacy

Respondents emphasized that training with digital technologies from various perspectives, such as engineering, law, data protection, and ethics as well as statistical knowledge in the context of evidence-based medicine, should be part of the curriculum. The digital literacy acquired in this way would include an understanding of the meaningfulness and application areas of AI, robotics, big data, and telemedicine and thus help in one's own clinical work and in cooperation with other professional groups. Increasingly, patients would use digital services and apps, but convincing doctors to use them would remain to be a major challenge. As health apps are already being used in practice, their medical, legal, ethical, and economic implications should be a part of medical education and training.

Digital literacy would describe the sensitivity, confidence, and understanding with which physicians could apply new digital applications to promote health. Respondents pointed out that by gaining practical experiences with digital health, medical school graduates should be able to use a wide variety of digital health technologies. Interpersonal skills such as intuition and sensory experience in patient contact would gain importance, especially regarding diagnostics. Health care staff would have to be able to communicate in various novel ways (eg, apps, telemedicine). Therefore, expertise in a variety of communication methods with a special emphasis on remote care should be acquired in medical schools.

##### Innovation Capabilities and Entrepreneurial Skills

Adapting to the constantly changing professional environment would require physicians to have their own inventive spirit and a lifelong willingness to progress and rethink. Physicians would be responsible for proactively shaping the transformation of medicine. This role could only be fulfilled by the corresponding conviction to be innovative. Innovation methods such as design thinking could be helpful to act visionary in this context.

Future medical school graduates should be trained to apply entrepreneurial skills in a digitalized and data-rich health care system to promote health care, according to the respondents. They should be able to think entrepreneurially and act innovatively, searching for new ways to sustainably integrate innovation into clinical workflows.

##### Transdisciplinary Cooperation and Open Access Mentality

Problems could be approached and solved confidently in transdisciplinary cooperation. The respondents stated that this should also be taken into account in the teaching concept for digital medicine. Cooperation with other disciplines, such as computer science, nursing, administration, user experience design, industry, law, data protection, and ethics, should be trained at an early stage because mutual understanding of the roles would facilitate communication.

In our health care system, including medical schools, much more cooperation and sharing of knowledge is needed. E-learning should ideally be publicly available.

##### Patient-Centered Approach

Physicians should be given the freedom to focus on patients rather than on technology. For this purpose, their focus should be on human interaction. For physicians, digital health would be about the ability to advise mature, proactive patients sensibly and to accompany them through the health care system appropriately through direct communication using digital health wherever appropriate.

##### Fearless of Change

Lack of awareness and experience in using digital technologies would fuel reservations in health care workers, according to the respondents. Actors, especially in leading positions, would feel that their professional authority or seniority was being questioned. Trying out and getting to know new possibilities in a relaxed manner could reduce fears, even among older generations and up to executive levels, and lead to successful clinical implementation.

#### Learning Objectives—Knowledge

##### Data Literacy

Clinical decisions were increasingly based on complex data. Identifying the relevant data and deriving good decisions from it would require a high degree of data competence from future physicians. Statistics would remain an important basis for scientific action in this context. It was stated that the responsible and sensible use of systems based on AI is an important medical skill and should become a compulsory learning objective for medical students.

##### Ethical, Legal, and Social Implications

Medicine in its current form would be unethical according to the respondents. Digitalization and modern data science could improve this by analyzing patient data to make clinical care more based on objective evidence. Often, issues in digital health would lead to ethical questions, which should be discussed by experts and students using exemplary situations. Legal aspects, in particular regarding the regulation of medical practice and medical devices, should be taught by experts during the course of studies. The culture of societal change in the context of digitalization should be a central component of the curriculum.

##### Data Protection

According to the interviewees, data protection, data security, and privacy were one of the greatest challenges for physicians in the course of digitalization, for example, in the case of mobile health apps. They should be taught transparently and according to clear guidelines by appropriate experts. As most patients care about the privacy of their health data, medical students should learn to ensure that their actions are in accordance with the correct data protection regulations.

##### Health Economics

Basic economic knowledge and health economic aspects such as financing and resource optimization were missing in medical schools’ curricula and should be taught specifically for digital health.

#### Teaching Format

##### Practical Experiences

The direct handling of new technologies would reduce fears and promote enthusiasm for digital technologies. *Learning by doing* would be the preferred form to learn about technical applications because it creates experiences, helps to shape interests, and ensures a *view beyond the end of one’s own nose* (interview quote). The goal of teaching digital health would be its successful clinical implementation. Therefore, existing technologies should be included in the medical curriculum and demonstrated by experienced lecturers. Students should be trained practically involving direct contact with patients and technologies to handle unexpected new situations in relation to digital health and encouraged to be creative and culturally reflective by creating their own content, for example, in the course of a hackathon.

##### Lectures

Short impulse lectures would be a useful teaching method, especially for large groups. Frontal lectures could be more effective for deepening a topic or as a supplement to practical training, especially if they do not last longer than 10 min.

##### Open Discourse

An open-ended discourse on the values, visions, and competencies of medical students among themselves and with various stakeholders in digital health (patients, physicians, nurses, etc) would lead to a high degree of reflection and heightened awareness of the relevance of the topic. Thus, critics and enthusiasts would be simultaneously involved.

##### Other Proposed Teaching Formats

Small group lessons in (carefully selected) groups of up to 10 students would ensure more enthusiasm for the course content, stated the respondents. Peer teaching would be very effective and bring individual experience into the curriculum. The creation of independent work is recommended as a teaching format. E-learning would be a suitable format, especially for theoretical content, and could be used in a scalable manner. In general, digital medicine teaching should be integrated longitudinally into the curriculum.

#### Cooperation

##### Cross-Faculty

Digital health could be taught as a study component of various study programs, such as medicine, economics, or sociology. According to respondents, transdisciplinary teaching was more attractive and stimulating. Interuniversity exchange would be profitable, and joint courses would be useful for students and lecturers.

##### Cross-Institutional and Cross-Functional

In the teaching of digital health, lecturers, students, scientists, alumni, and experts from all participating study programs as well as external experts would be partners. It would also be advisable for the lecturers to consult with each other to ensure continuity and interlocking of the teaching content.

##### Industry and Business

The respondents believe that integration of different actors from industry and business into the teaching of digital health at medical schools would help both students and companies to benefit and learn from each other.

### Quantitative Results

#### Students’ Evaluation

**Figure 2:**
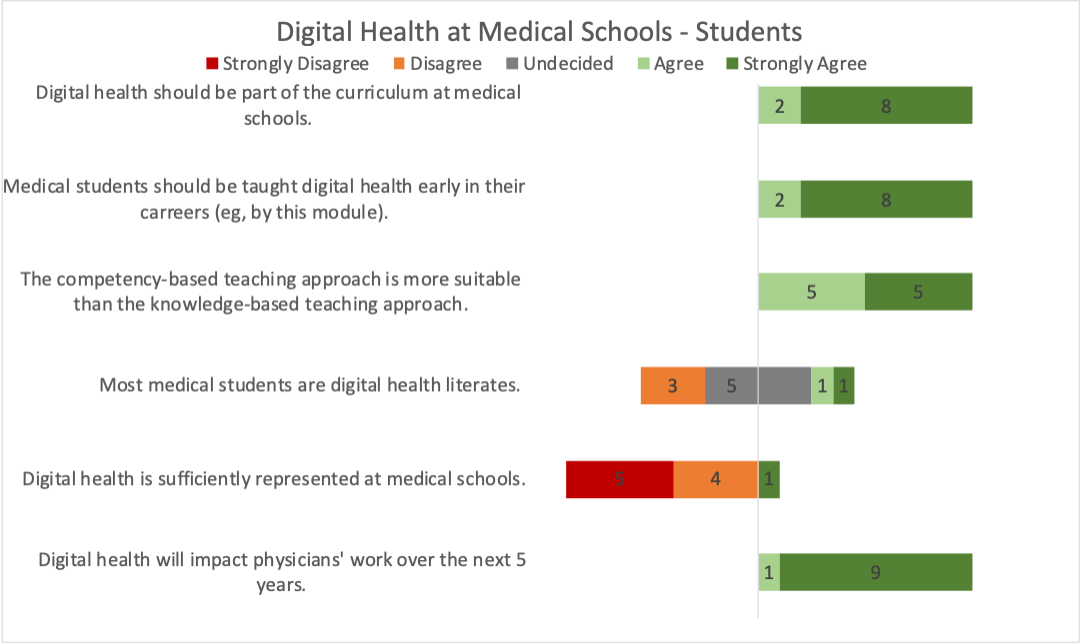
Students’ opinions regarding digital health at medical schools.

All participating students agreed that digital health should be part of the mandatory curriculum at medical schools (8 strongly agree and 2 agree) and taught early in their careers (8 strongly agree and 2 agree) and that competencies are more relevant than knowledge for medical graduates (5 strongly agree and 5 agree; Figure 2). Regarding whether medical students are digitally literate, the opinion was split. Most students strongly disagreed or disagreed that digital health is sufficiently represented at medical schools (5 strongly disagree, 4 disagree, and 1 agree). All students agreed that digital health would impact physicians’ work over the next 5 years (9 strongly agree and 1 agree).

**Figure 3:**
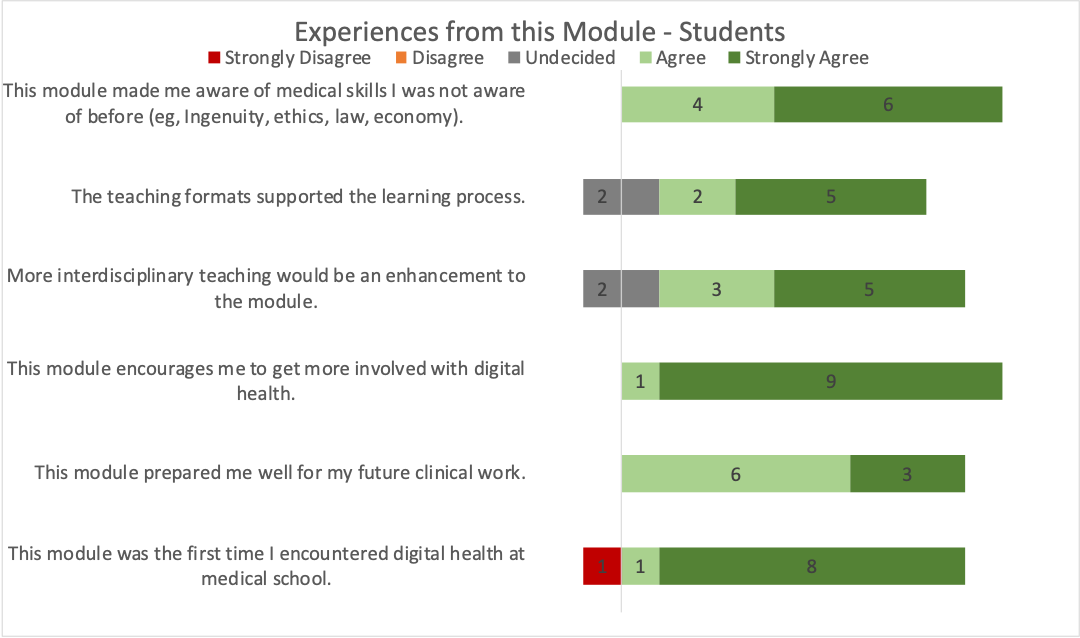
Students’ experience with the digital health module.

Regarding the experiences from this module, all students agreed that this module made them aware of medical skills that they were not aware of before (6 strongly agree and 4 agree), that the teaching format helped them in the study process (5 strongly agree, 2 agree, and 2 neutral), and that interdisciplinary teaching formats would be an enhancement to the module (5 strongly agree, 3 agree, and 2 neutral; Figure 3). All students agreed that the module motivated them to get more involved with digital health (9 strongly agree and 1 agree) and that they felt well prepared for their future clinical work (3 strongly agree and 6 agree). For most students, this module was the first time they encountered digital health at medical school (8 strongly agree, 1 agree, and 1 strongly disagree).

There was a strong overall satisfaction with the organizational aspects of the module (Multimedia Appendix 3).

#### Lecturers’ Evaluation

**Figure 4:**
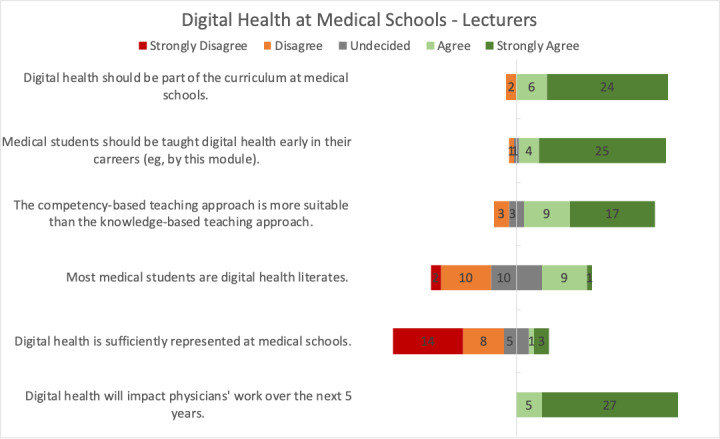
Lecturers’ opinions regarding digital health at medical schools.

The majority of the lecturers stated that digital health should be part of the mandatory curriculum at medical schools (24 strongly agree, 6 agree, and 2 disagree) and that medical students should be taught digital health early in their career (25 strongly agree, 4 agree, 1 neutral, and 1 disagree; Figure 4). Most lecturers responded that a competency-centered teaching approach is more suitable than a knowledge-based approach (17 strongly agree, 9 agree, 3 neutral, and 3 disagree). Regarding digital literacy among medical students, opinions were split. The majority of lecturers disagreed that digital health would be sufficiently represented at medical schools (14 strongly disagree, 8 disagree, 5 neutral, 1 agree, and 3 strongly agree). All lecturers agreed that digital health will impact physicians’ work over the next 5 years (27 strongly agree and 5 agree).

**Figure 5:**
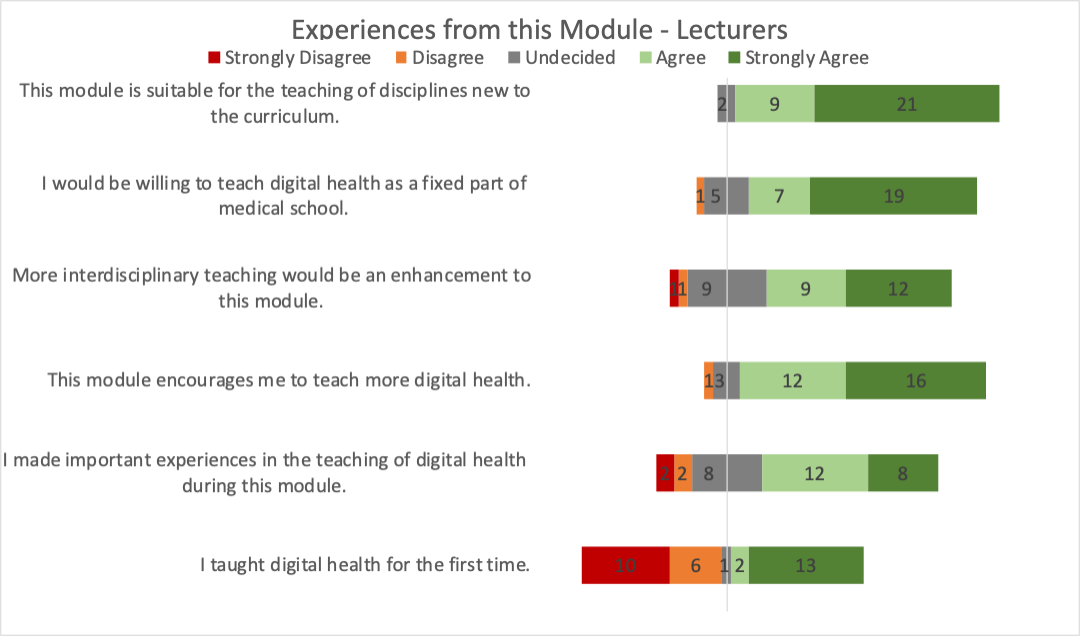
Lecturers’ experience with teaching in the digital health module.

Regarding experiences from this module, lecturers agreed that this module over 3 weeks is suitable for teaching new disciplines (21 strongly agree, 9 agree, and 2 neutral; Figure 5). The majority of lecturers agreed that they would be willing to teach digital health as a fixed part of the medical curriculum (19 strongly agree, 7 agree, 5 neutral, and 1 disagree) and that more interdisciplinary teaching would enhance this module (12 strongly agree, 9 agree, 9 neutral, 1 disagree, and 1 strongly disagree). Lecturers stated that this module animated them to teach more about digital health (16 strongly agree, 12 agree, 3 neutral, and 1 disagree). Regarding whether lecturers made important experiences during this module as well as whether lecturers taught digital health for the first time, opinions were split.

Overall, there was a high satisfaction rate for module organization, although in some individual cases, room for improvement was seen concerning the organizational communication (Multimedia Appendix 3).

## Discussion

### Principal Findings

In this mixed methods study, we provide valuable evidence on digital health teaching at medical schools. Our findings indicate that teaching digital health in medical schools not only comprises the knowledge transfer of technological advancements in health care. However, equally important is to shape the mindset of future physicians, teaching them to be open toward innovation, willing to develop and share novel insights through a transdisciplinary and patient-centered approach, and to be able to overcome fears of adopting digital health technology in clinical routine. Our results show that practical experiences, for example, with clinically implemented technology, bedside teaching, or by own creations as hackathons, are fundamental and should only be complemented with short impulse lectures. Small groups and peer teaching support the learning process.

The vast majority of lecturers and students stated that digital health is not sufficiently represented at medical schools and should be part of the curriculum. Both lecturers and students felt encouraged to get more involved with digital health after participating in the module.

### Need for Digital Health

The digital transformation of our health care system can increase the quality, accessibility, and affordability of health services [29]. By implementing digital technology for health care provision, we can apply health services more efficiently and individually. Within the next 20 years, it is expected that 90% of all jobs in the United Kingdom’s National Health Service would require digital skills [30]. With an increasing flood of information for medical staff, there are already data reporting staff to be overwhelmed by alarms and notifications [31]. Intelligent systems may filter these and other digital information, making them better usable for patient care, which is more urgent than ever in some medical disciplines [32]. Furthermore, many processes could be simplified through the implementation of telemedicine in health care, thereby optimizing patient care [33,34].

Some sources name a lack of health care professionals’ awareness and knowledge as a main barrier to a successful digital transformation of health systems [30,35]. The European Medical Students’ Association recently identified a significant gap between students’ willingness to act in a digitalized health care environment and the knowledge and skills acquired in their curriculum [23,24,36]. Medical staff and medical students can be regarded as ready and willing to use digital health technology in their clinical routine. However, adequate and regular training in digital devices as well as a structured curriculum in digital health are still lacking in most institutions [37,38], whereas some associations recommend up to 40 hours of education on biomedical and health informatics for medical and nursing students during their studies [39]. Our findings show that the need for digital health and its teaching is increasing, which is acknowledged by students, lecturers, and experts. Therefore, mandatory modules on digital health, medical informatics, or medical technologies should be included in the curricula of medical schools.

### Digital Health in Medical Schools

Elective classes and programs on digital health slowly find their way into medical curricula. This process is driven by individual pilot projects rather than by validated and coordinated guidelines or national regulations [16,38,40-48]. Pilot projects, adapted to the individual curricular conditions of medical faculties, can be the first step toward realizing a longitudinal interdisciplinary approach to implement digital health in the overall curriculum [13,16,49-51].

In 2013, the German Association for Medical Informatics, Biometry and Epidemiology developed national competency–based learning objectives titled *Medical Informatics Learning Objectives for Undergraduate Medical Education*, which are consistent with the recommendations of the International Medical Informatics Association [50]. The objectives are in line with our findings implicating that medical graduates should have a broad knowledge of data literacy; be trained to assess ethical, legal, and social implications (ELSIs) and privacy aspects of digital health; and estimate the health economic consequences of novel technologies. Here, specialist expertise in digitalization beyond medicine could be offered in a local interdisciplinary and interprofessional network of universities. Even impressions from politics and business should be included to convey a comprehensive picture. Finally, addressing a peer-to-peer teaching approach, the motivation and competencies of many medical students as digital natives may have a very high potential to be included in medical curricular teaching offerings.

The national and international exchange of *best practice* examples and networking will be helpful in this respect. At the same time, however, urgent efforts should be made to define standards for the teaching of digital skills in medical studies at national levels [39].

### Undergraduate Medical Competencies in Digital Health

The future-proof physician must be able to apply digital health in the clinical routine. Our results emphasize that physicians should be open toward innovation and transdisciplinary cooperation throughout their careers and include the ability to recognize entrepreneurial potentials. Brunner et al [38] classified the digital health capabilities expected of medical graduates into 4 domains: (1) *digital technologies, systems, and policies*, covering digital literacy and ELSI; (2) c*linical practice and applications*, including the ability to integrate digital health into clinical routine; (3) *data analysis and knowledge creation,* including the ability to apply basic data analytics to unstructured digital data sets; and (4) and *system and technology implementation,* suggesting that medical professionals should participate in the development and implementation of digital health. The latter aspect is also stressed by our results and a recent publication that demands physicians with dual competencies in clinical and data science expertise [49]. A medical graduate should be able to use digital health technology, interpret its results, and explain those to the patients.

Another main finding of our study is that teaching digital health should be about teaching a mindset that allows flexibility and suitability for an unknown and rapidly evolving future of our health care sector, rather than teaching the use of individual technologies or the right approach only for specific situations. This mindset includes openness to change, curiosity, and collaboration across functions, roles, professions, institutions, and hierarchical gradients. It also requires the willingness to actively anticipate and form the future of health care through reflection and innovation. Eventually, a strong awareness of changing patients’ and health care workers’ needs and for new ELSIs should result from this mindset. It may be acquired by confrontation with a broad variety of different digital health aspects in terms of patient treatment, medical technologies, and the structure of health systems, as we did in our module.

Our results emphasize that passive knowledge transfer in the form of lectures is outdated, although it is the most used teaching method in medical schools [52]. A focus on students’ engagement (eg, own creation in hackathons, peer teaching) and linking knowledge transfer to practical experiences (eg, bedside teaching with available digital technology) is desirable and effective. Students should be given the opportunity to deepen their knowledge and skills in special fields (eg, clinical data science, design thinking) in extracurricular activities or in the form of an elective module [49]. The exchange with other disciplines in workshops or hackathons can be the seed for future health innovations and entrepreneurial ventures. They are promising methods for introducing the inventor mindset to future physicians and offer the possibility of teaching medical students together with students of more information technology (IT)–focused and data-focused subjects. A close collaboration with IT faculties, for example, through an MD and computer scientist tandem, should be anticipated.

### Limitations

This mixed methods study provided novel insights into undergraduate medical competencies in light of digital transformation in health care and the development and deployment of a digital health module at a German medical school. However, a number of limitations apply.

First, the results are based on a limited number of participants. As a descriptive approach, quantification or generalization of the results is not possible but may still provide important impulses for further research and development in the field. Second, due to the selection bias of students and lecturers, the results of the questionnaire might only reflect the opinion of digital health advocates and are not representative. Third, the described undergraduate competencies in digital health are an additional source of information; therefore, there is no guarantee of completeness.

### Conclusions

Digital health competencies are key competencies for future-proof physicians and should therefore be included in the core curriculum at medical schools. For a first curricular implementation and to gain early local experiences, elective modules such as ours are suitable and can provide important information for existing didactic strengths in teaching this topic. The exchange of best teaching practices and institutional collaborations will further leverage the reach of this intent.

Digital health education should focus on building and sharpening a critical and experience-based *digital health mindset*, rather than passively transferring knowledge of technology specifics. Active teaching methods such as practice units, discussions with experts, and hackathons should be used to teach a broad variety of subjects. Interprofessional learning and teaching with nonmedical disciplines is recommended to add to the diversity of perspectives and to prepare for increasingly interprofessional patient care.

## Appendix

Multimedia Appendix 1 Time table.

Multimedia Appendix 2 Survey questionnaires.

Multimedia Appendix 3 Survey questionnaire results.

## Abbreviations

AI: artificial intelligence
ELSI: ethical, legal, and social implications
IT: information technology

## References

[1] Wald HS, George P, Reis SP, Taylor JS (2014). Electronic health record training in undergraduate medical education: bridging theory to practice with curricula for empowering patient- and relationship-centered care in the computerized setting. Acad Med.

[2] Bundesgesundheitsministerium. Die Elektronische Gesundheitskarte.

[3] Tierney MJ, Pageler NM, Kahana M, Pantaleoni JL, Longhurst CA (2013). Medical education in the electronic medical record (EMR) era: benefits, challenges, and future directions. Acad Med.

[4] Esteva A, Kuprel B, Novoa RA, Ko J, Swetter SM, Blau HM, Thrun S (2017). Dermatologist-level classification of skin cancer with deep neural networks. Nature.

[5] Magrabi F, Ammenwerth E, McNair JB, De Keizer NF, Hyppönen H, Nykänen P, Rigby M, Scott PJ, Vehko T, Wong ZS, Georgiou A (2019). Artificial intelligence in clinical decision support: challenges for evaluating AI and practical implications. Yearb Med Inform.

[6] Waseh S, Dicker AP (2019). Telemedicine training in undergraduate medical education: mixed-methods review. JMIR Med Educ.

[7] Pathipati AS, Azad TD, Jethwani K (2016). Telemedical education: training digital natives in telemedicine. J Med Internet Res.

[8] Krüger-Brand HE Telemedizin: Vor dem Durchbruch. Deutsches Ärzteblatt.

[9] Uslu Y, Altınbaş Y, Özercan Tuğba, van Giersbergen MY (2019). The process of nurse adaptation to robotic surgery: a qualitative study. Int J Med Robot.

[10] Greenhalgh T, Wherton J, Shaw S, Morrison C (2020). Video consultations for covid-19. Br Med J.

[11] Fong DJ Patient-centric Technology Improves Access, Efficiency, and Quality of Care | Clinical Drug Information Internet. Wolters Kluwer.

[12] Digitalisierung im Gesundheitswesen: die 34-Milliarden-Euro-Chance für Deutschland.

[13] Kuhn S, Kadioglu D, Deutsch K, Michl S (2018). Data literacy in der medizin. Onkologe.

[14] (2018). #SmartHealthSystems. Bertelsmann-Stiftung.

[15] Ross J, Stevenson F, Lau R, Murray E (2016). Factors that influence the implementation of e-health: a systematic review of systematic reviews (an update). Implement Sci.

[16] Chandrashekar P (2019). A digital health preclinical requirement for medical students. Acad Med.

[17] Ridsdale C, Rothwell J, Smit M, Ali-Hassan H, Bliemel M, Irvine D, Kelley D, Matwin S, Wuetherick B (2015). Strategies and Best Practices for Data Literacy Education: Knowledge Synthesis Report Internet. Semantic Scholar.

[18] Loda T, Erschens R, Junne F, Stengel A, Zipfel S, Herrmann-Werner A (2020). Undergraduate medical students' search for health information online: explanatory cross-sectional study. JMIR Med Inform.

[19] Smith EE, Kahlke R, Judd T (2020). Not just digital natives: integrating technologies in professional education contexts. Australas J Educ Technol.

[20] Pontefract SK, Wilson K (2019). Using electronic patient records: defining learning outcomes for undergraduate education. BMC Med Educ.

[21] Blakemore LM, Meek SE, Marks LK (2020). Equipping learners to evaluate online health care resources: longitudinal study of learning design strategies in a health care massive open online course. J Med Internet Res.

[22] de Araújo Novaes M, Sá de Campos Filho A, Diniz PR (2019). Improving education of medical students through telehealth. Stud Health Technol Inform.

[23] Mosch L EMSA Survey on EHealth Presentation Deans Meeting Rotterdam Internet. European Medical Students' Association.

[24] Machleid F, Kaczmarczyk R, Johann D, Balčiūnas J, Atienza-Carbonell B, von Maltzahn F, Mosch L (2019). Perceptions of Digital Health Education Among European Medical Students: Mixed Methods Survey.

[25] (2019). European Deans Meeting: Training Future-Proof Doctors for the Digital Society. European Medical Students' Association.

[26] Dubois A, Gadde L (2002). Systematic combining: an abductive approach to case research. J Bus Res.

[27] Harris PA, Taylor R, Minor BL, Elliott V, Fernandez M, O'Neal L, McLeod L, Delacqua G, Delacqua F, Kirby J, Duda SN, REDCap Consortium (2019). The REDCap consortium: building an international community of software platform partners. J Biomed Inform.

[28] Harris PA, Taylor R, Thielke R, Payne J, Gonzalez N, Conde JG (2009). Research electronic data capture (REDCap)--a metadata-driven methodology and workflow process for providing translational research informatics support. J Biomed Inform.

[29] WHO Guideline: Recommendations on Digital Interventions for Health System Strengthening. World Health Organization.

[30] Lennon MR, Bouamrane M, Devlin AM, O'Connor S, O'Donnell C, Chetty U, Agbakoba R, Bikker A, Grieve E, Finch T, Watson N, Wyke S, Mair FS (2017). Readiness for delivering digital health at scale: lessons from a longitudinal qualitative evaluation of a national digital health innovation program in the United Kingdom. J Med Internet Res.

[31] Bach TA, Berglund L, Turk E (2018). Managing alarm systems for quality and safety in the hospital setting. BMJ Open Qual.

[32] Komorowski M (2020). Clinical management of sepsis can be improved by artificial intelligence: yes. Intensive Care Med.

[33] Koehler F, Koehler K, Deckwart O, Prescher S, Wegscheider K, Kirwan B, Winkler S, Vettorazzi E, Bruch L, Oeff M, Zugck C, Doerr G, Naegele H, Störk S, Butter C, Sechtem U, Angermann C, Gola G, Prondzinsky R, Edelmann F, Spethmann S, Schellong SM, Schulze PC, Bauersachs J, Wellge B, Schoebel C, Tajsic M, Dreger H, Anker SD, Stangl K (2018). Efficacy of telemedical interventional management in patients with heart failure (TIM-HF2): a randomised, controlled, parallel-group, unmasked trial. Lancet.

[34] Koenig M (2019). Telemedicine in the ICU.

[35] Gagnon M, Ngangue P, Payne-Gagnon J, Desmartis M (2016). m-Health adoption by healthcare professionals: a systematic review. J Am Med Inform Assoc.

[36] Machleid F, Kaczmarczyk R, Johann D, Balčiūnas J, Atienza-Carbonell B, von Maltzahn F, Mosch L (2020). Perceptions of digital health education among European medical students: mixed methods survey. J Med Internet Res.

[37] Poncette A, Spies C, Mosch L, Schieler M, Weber-Carstens S, Krampe H, Balzer F (2019). Clinical requirements of future patient monitoring in the intensive care unit: qualitative study. JMIR Med Inform.

[38] Brunner M, McGregor D, Keep M, Janssen A, Spallek H, Quinn D, Jones A, Tseris E, Yeung W, Togher L, Solman A, Shaw T (2018). An ehealth capabilities framework for graduates and health professionals: mixed-methods study. J Med Internet Res.

[39] Haag M, Igel C, Fischer MR, German Medical Education Society (GMA)‚ Committee Digitization – Technology-Assisted LearningTeaching, Joint working group Technology-enhanced Teaching and Learning in Medicine (TeLL) of the German Association for Medical Informatics‚ BiometryEpidemiology (gmds)the German Informatics Society (GI) (2018). Digital teaching and digital medicine: a national initiative is needed. GMS J Med Educ.

[40] Timothy DA, Ravi P Integrating digital health into the curriculum: considerations on the current landscape and future developments. Sage Publications.

[41] Gray K, Dattakumar A, Maeder A, Butler-Henderson K, Chenery H (2014). Advancing Ehealth Education for the Clinical Health Professions: Final Report.

[42] Digital Health Internet. UC San Diego Extension.

[43] Academic Programs. Thomas Jefferson University.

[44] (2017). Certificate in Digital Health Communication. Tufts Public Health.

[45] Fernando J, Lindley J (2018). Lessons learned from piloting mHealth informatics practice curriculum into a medical elective. J Am Med Inform Assoc.

[46] Justus-Liebig-Univ Gießen. SPC Digitale Medizin, eHealth und Telemedizin.

[47] Mesko B, Győrffy Z, Kollár J (2015). Digital literacy in the medical curriculum: a course with social media tools and gamification. JMIR Med Educ.

[48] Prodekanat für Lehre - Modellstudiengang iMED. UKE.

[49] McCoy LG, Nagaraj S, Morgado F, Harish V, Das S, Celi LA (2020). What do medical students actually need to know about artificial intelligence?. NPJ Digit Med.

[50] Röhrig R, Stausberg J, Dugas M, GMDS project group Medical Informatics Education in Medicine” (2013). Development of national competency-based learning objectives 'medical informatics' for undergraduate medical education. Methods Inf Med.

[51] Offergeld C, Neudert M, Emerich M, Schmidt T, Kuhn S, Giesler M (2020). [Mediation of data literacy in curricular education in otorhinolaryngology: watch and wait or anticipatory obedience?]. HNO.

[52] Dickman N, Schuster B (2020). Active Education for Future Doctors.

