# Correction: Undergraduate Medical Competencies in Digital Health and Curricular Module Development: Mixed Methods Study

**DOI:** 10.2196/25738

**Published:** 2020-12-07

**Authors:** Akira-Sebastian Poncette, Daniel Leon Glauert, Lina Mosch, Katarina Braune, Felix Balzer, David Alexander Back

**Affiliations:** 1 Department of Anesthesiology and Intensive Care Medicine Charité – Universitätsmedizin Berlin Corporate member of Freie Universität Berlin, Humboldt-Universität zu Berlin, and Berlin Institute of Health Berlin Germany; 2 Einstein Center Digital Future Berlin Germany; 3 Department of Paediatric Endocrinology and Diabetes Charité – Universitätsmedizin Berlin Corporate member of Freie Universität Berlin, Humboldt-Universität zu Berlin, and Berlin Institute of Health Berlin Germany; 4 Department of Traumatology and Orthopaedics, Septic and Reconstructive Surgery Bundeswehr Hospital Berlin Berlin Germany; 5 Dieter Scheffner Center for Medical Education and Educational Research Charité – Universitätsmedizin Berlin Corporate member of Freie Universität Berlin, Humboldt-Universität zu Berlin, and Berlin Institute of Health Berlin Germany

In “Undergraduate Medical Competencies in Digital Health and Curricular Module Development: Mixed Methods Study” (J Med Internet Res 2020;22(10):e22161) the authors noted three errors.

One author's name was displayed as:

David Back

It has now been corrected to:

David Alexander Back

One affiliation for David Alexander Back was listed as:

Bundeswehr Hospital Berlin, Clinic for Traumatology and Orthopedics, Septic-Reconstructive Surgery, Berlin, Germany

It has now been corrected to:

Department of Traumatology and Orthopaedics, Septic and Reconstructive Surgery, Bundeswehr Hospital Berlin, Berlin, Germany

The phone number for corresponding author was incorrectly listed. It has been changed to:

49 30 2841 1240

The correction will appear in the online version of the paper on the JMIR Publications website on December 7, 2020, together with the publication of this correction notice. Because this was made after submission to PubMed, PubMed Central, and other full-text repositories, the corrected article has also been resubmitted to those repositories.

