# Supplementary material for: Undergraduate Medical Competencies in Digital Health and Curricular Module Development: Mixed Methods Study
Source: J Med Internet Res. 2020 Oct 29;22(10):e22161. doi: 10.2196/22161 (PMC7661229; doi:10.2196/22161)
Supplement: Multimedia Appendix 1 [file jmir_v22i10e22161_app1.docx]

**Timetable Elective module (M24)**

**"Digital Health: From apps to the smart hospital"**

**1st week (total: 19 teaching units, large group: 13 teaching units, small group: 6 teaching units)**

| **Monday** | **Tuesday** | **Wednesday** | **Thursday** | **Friday** |  |
| --- | --- | --- | --- | --- | --- |
| **20.01.20 (CCM)** | **21.01.20 (CCM)** | **22.01.20 (BwK)** | **23.01.20 (CCM)** | **24.01.20 (CVK)** |  |
| **08:30 – 09:15**  **Welcome and introduction to the module with explanations of structural and content specifics** (large group, 45min)  CMSC & Anesthesiology | **08:30 – 10:00**  **Possible applications of the mobile phone in dermatology** (small group, 2x45min)  Dermatology | **8:30-10:00**  **3D digital and real: Indications and applications of** **3D reconstructions via CT and 3D printing for surgery planning**  (small group, 2x45min) BwKrhs Berlin (with CMSC) | **08:30 – 09:15**  **History and basics of telecardiology** (large group, 45min) Telecardiology | **08:30 – 10:00**  **Digital Smart Implants and their applications in basic and clinical research** (large group, 2x45min)  Julius Wolff Institute |  |
| **09:30 – 10:15**  **Introduction to the basics of Digital Health** (large group, 45min)  CMSC & Anesthesiology | **10:15 – 11:00**  **Legal bases of digitisation - where are we and where are we going?** (Large group, 45min) Medical law (Dierks+Company) | **10:00-10:45**  **Start up - opportunities and risks**  (large group, 45min)  BwKrhs Berlin (with CMSC) | **09:15 – 10:00**  **Potential and practical application of telecardiology based on clinical examples** (large group, 45min) Telecardiology | **10:15 – 11:00**  **Insoles and wearables in movement measurement (day 1) - practical testing and instruction in use (day 2 then in the 3rd week)** (small group, 45min)  Julius Wolff Institute |  |
| **10:30 – 11:15**  **Teambuilding with preparation for individual small group work**  (large group, 45min) Anaesthesiology (with Hacking Health Berlin and Hasso Plattner Institute) | **11:15 – 12:00**  **Guidelines for mHealth: Do we need a TÜV for Apps?**  (large group, 45min)  Medical Law (Dierks+Company) CMSC & Anaesthesiology | **10:45-12:15**  **The potential of artificial intelligence using the example of symptom checkers** (large group, 90min) CMSC with ADA Health | **10:15 – 11:00**  **„Digital Health for Cardiology “**  **(Day 1) - Introduction to testing apps and wearables for recording cardio parameters (Day 2 in the 3rd week)** (small group, 45min) Anesthesiology & CMSC (Hasso Plattner Institute) | **11:30 – 13:00**  **Presentation of a tele-intensive care unit**  (large group, 2x45min) Anesthesiology |  |
|  |  |  | **11:15 – 12:15**  **Clinical decision support - potentials and challenges**  (large group, 45min) Anesthesiology |  |  |

CMSC = Centre for Musculoskeletal Surgery, Dierks+Company Rechtsanwaltsgesellschaft mbH, BwKrhs = Bundeswehrkrankenhaus Berlin

| **2nd week (total: 22 teaching units, large group: 14 teaching units, small group: 8 teaching units)** | | | | | | | | |  |  | | |  | |  | | |  |
| --- | --- | --- | --- | --- | --- | --- | --- | --- | --- | --- | --- | --- | --- | --- | --- | --- | --- | --- |
| **Monday** | | **Tuesday** | **Wednesday** | | **Thursday** | | | **Friday** | | | |  | |  |  |  |  |  |
| **27.01.20 (CVK)** | | **28.01.20 (CCM)** | **29.01.20 (CVK)** | | **30.01.20 (CCM)** | | | **31.01.20 (CCM)** | | | |  | |  |  |  |  |  |
| **08:30 – 09:15**  **Digital applications in the branch office (e-doctor's letter, online consultation hours, patient management)** (large group, 45min)  Interdisciplinary Joint Practice Schlüterstraße with CMSC | | **08:30 – 09:15**  **Routine data and use in health economics and health system research**  (large group, 45min) Health economics | **08:30 – 09:15**  **Basics Mixed/Virtual/Augmented Reality (case studies and device briefing)**  (large group, 45min)  Surgery | | **08:30 – 09:15** Randomized controlled studies with mHealth Apps - lessons learned (large group, 45min) Health economics | | | **08:30 – 10:00**  **Value-based Radiology and the Digital Revolution - How digitization influences the potential of radiology** (large group, 2x45min) Radiology | | | |  | |  |  |  |  |  |
| **09:45 – 11:15**  **Experience of using an online video consultation**  (large group, 2x45min) Pediatrics, Neurosurgery & CMSC | | **09:30 – 11:45**  **Introduction and practice: Charité Mini-Hackathon**  **(Part 1, Problem solving)** (small group, 3x45min)  Anesthesiology & Pediatrics (and Hacking Health Berlin, Hasso-Plattner-Institute) | **09:30 – 12:30**  **Digital surgical circuit training** **1. mixed reality in anatomy and surgical preparation**  **2. application of mixed reality in the operating theatre**  **3. use of virtual reality for OP training** **4. presentation of the function and possibilities of the DaVinci** (small group, 4x45min)  Surgery & CMSC | | **09:30 – 11:00**  **Ethical challenges of digital medicine**  (large group, 2x45min) Ethics | | | **10:15 – 11:45**  **Digital health in psychiatry** (large group, 2x45min)  Psychiatry | | | |  | |  |  |  |  |  |
|  | | **12:00 – 12:45**  **Big Data and AI: Terms, basics, applications and potentials** (large group, 45min)  Surgery (Lohmann) |  | | **11:15 – 12:00**  **Personalized drug therapy - digital medicines, personal drugs & Co** (large group, 45min)  Clinical Pharmacology | | |  | | | |  | |  |  |  |  |  |
|  | |  |  | | **12:00 – 12:45**  **Software systems and wise decision making in drug prescription - "Choosing wisely**  (small group, 45min)  Clinical Pharmacology | | |  | | | |  | |  |  |  |  |  |
|  |  |  |  |  |  |  |  |  |  |  |  |  |  |  |  |  |  |  |
| CMSC = Center for Musculoskeletal Surgery, BwKrhs = Bundeswehrkrankenhaus Berlin  **3rd week (total: 19 teaching units, large group: 11 teaching units, small group: 8 teaching units)** | | | |  | |  | | |  | |  | | | | |  |  |  |
| **Monday** | | **Tuesday** | **Wednesday** | **Thursday** | | | **Friday** | | | |  | | |  |  |  |  |  |
| **03.02.20 (EGZB)** | | **04.02.20 (HPI)** | **05.02.20 (CVK)** | **06.02.20 (CCM)** | | | **07.02.20** | | | |  | | |  |  |  |  |  |
| **08:30 – 09:15**  **Fall prophylaxis and robotics in old age** (large group, 45min)  Geriatrics | | **08:30 – 09:15**  **"Digital Health for Cardiology" (day 2) - evaluation of cardiac parameters** (large group, 45min)  Anesthesiology & CMSC  (Hasso Plattner Institute) | **08:30 – 09:15**  **Digitization and data management in the hospital of the future**  (large group, 45min) Chief Digital Officer of the Charité | **08:30 – 10:15**  **Practice: Charité Mini-Hackathon**  **(Part 3, Presentation skills)** **Hacking Health Berlin** (small group, 3x45min)  Anesthesiology & Pediatrics (with Hacking Health Berlin, Hasso-Plattner-Institute) | | | **Free** | | | |  | | |  |  |  |  |  |
| **09:15 – 10:00**  **Use of virtual reality for chronic back pain and arterial hypertension**  (small group, 45min)  Geriatrics | | **09:30 – 10:15**  **The basics about mobile Health (mHealth) – more than just a smartphone app** (Großgruppe, 45min)  Anesthesiology & CMSC  (with Hasso Plattner Institute) | **09:30 – 10:15**  **Application of Data Driven Design and parametric design methods in the planning of hospitals**  (large group, 45min) CMSC (with HDR GmbH) | **10:30 – 11:15**  **Evaluation Charité Mini-Hackathon** (large group, 45min)  Anesthesiology & Pediatrics (with Hacking Health Berlin, Hasso-Plattner-Institute) | | |  | | | |  | | |  |  |  |  |  |
|  | | **10:30 – 13:00**  **Practice: Charité Mini-Hackathon (Part 2, Prototyping)**  **Hacking Health Berlin**  (small group, 4x45min)  Anesthesiology & Pediatrics (with Hacking Health Berlin, Hasso-Plattner-Institute) | **10:30 – 11:15**  **Insoles and wearables in movement measurement (day 2) - evaluation of steps/movement patterns** (large group, 45min) | **11:15 – 12:00**  **Quiz, evaluation and completion of the compulsory elective module**  (large group, 2x45min)  CMSC & Anesthesiology | | |  | | | |  | | |  |  |  |  |  |
|  | | **Optional: 14:00 - 17:00**  **Practice: Charité Mini-Hackathon (continuation of part 2, prototyping)**  **Hacking Health Berlin**  Anesthesiology & Pediatrics (with Hacking Health Berlin, Hasso-Plattner-Institute) | **11:30 – 12:15**  **Discussion of the experiences with the own data recording on the topic "transparent patients and doctors?**  (large group, 45min) CMSC & Anesthesiology |  | | |  | | | |  | | |  |  |  |  |  |

CMSC = Centre for Musculoskeletal Surgery, HDR GmbH = International Architecture Office, HPI = Hasso Plattner Institute Potsdam, Digital Health Center
