# Supplementary material for: Undergraduate Medical Competencies in Digital Health and Curricular Module Development: Mixed Methods Study
Source: J Med Internet Res. 2020 Oct 29;22(10):e22161. doi: 10.2196/22161 (PMC7661229; doi:10.2196/22161)
Supplement: Multimedia Appendix 2 [file jmir_v22i10e22161_app2.pdf]

Dozierendenbefragung zum Wahlpflichtmodul "Digital Health"

Sehr geehrte Dozierende,

bitte geben Sie uns Rückmeldung zu Ihrer Erfahrung mit unserem gemeinsamen Wahlpflichtmodul "Digital Health". Ihr Feedback wird uns bei der wissenschaftlichen Auswertung und Verbesserung der Lehre helfen. Die Ergebnisse werden anonymisiert im Rahmen unseres Projekts „IDHEC“ (Implementation of a Digital Health Elective Class at a German Medical School) veröffentlicht. Die Ethikkommission der Charité hat dieser Befragung zugestimmt (EA Nummer EA1/236/19).

Im Namen des gesamten Organisationsteams und der Studierenden bedanken wir uns noch einmal recht herzlich für Ihr Engagement und die Zusammenarbeit!

Daniel Glauert, Doktorand, Charité daniel-leon.glauert@charite.de

PD Dr. David Back, Studienleitung, BwKrhs Berlin, DSFZ Charité, david.back@charite.de

Dr. Akira-Sebastian Poncette, Prof. Dr. Dr. Felix Balzer, Studienkoordinatoren, Klinik für Anästhesiologie m.S. operative Intensivmedizin Charité

Organisation des Wahlpflichtmoduls

		Stimme voll und ganz zu	Stimme zu	Neutral	Stimme nicht zu	Stimme ganz und gar nicht zu
1)	Ich war mit der Organisation des Moduls zufrieden.	○	○	○	○	○
2)	Ich war mit der Themenzusammenstellung des Moduls zufrieden.	○	○	○	○	○
3)	Ich war mit dem Interesse und der Beteiligung der Studierenden zufrieden.	○	○	○	○	○
4)	Ich war mit der Kommunikation mit dem Organisationsteam zufrieden.	○	○	○	○	○
5)	Ich hatte genug Zeit zur Vermittlung der von mir geplanten Aspekte.	○	○	○	○	○

6) Freie Kommentare zu diesem Unterthema (Organisation des Wahlpflichtmoduls): ______________________

Erfahrungen aus dem Wahlpflichtmodul

		Stimme voll und ganz zu	Stimme zu	Neutral	Stimme nicht zu	Stimme ganz und gar nicht zu
7)	Ich habe in diesem Rahmen erstmals zu Aspekten der digitalen Medizin gelehrt.	○	○	○	○	○
8)	Ich konnte wichtige Erfahrungen für die Lehre von digitalen Medizininhalten sammeln.	○	○	○	○	○
9)	Ich fühle mich bestärkt, in Zukunft mehr Lehre zu digitaler Medizin zu betreiben.	○	○	○	○	○
10)	Ich würde noch mehr interdisziplinäre Lehrveranstaltungen für sinnvoll halten. (Falls Sie zustimmen, bitte erläutern Sie unten im Kommentarfeld.)	○	○	○	○	○
11)	Ich wäre bereit, die in diesem Wahlpflichtmodul gelehrten Aspekte der digitalen Medizin auch im regulären Curriculum allen Medizinstudierenden anzubieten.	○	○	○	○	○
12)	Das Wahlpflichtmodul ist zur Erprobung neuer Lehrinhalte im Kontext von digitaler Medizin geeignet.	○	○	○	○	○

13) Freie Kommentare zu diesem Unterthema (Erfahrungen aus dem Wahlpflichtmodul):

____

Digitale Medizin im Medizinstudium

	Stimme voll und ganz zu	Stimme zu	Neutral	Stimme nicht zu	Stimme ganz und gar nicht zu
14) Digitale Medizin wird den Berufsalltag von ÄrztInnen in den nächsten 5 Jahren stark beeinflussen.	○	○	○	○	○
15) Digitale Medizin ist im aktuellen Medizinstudium aus meiner bisherigen Erfahrung ausreichend und gut abgebildet.	○	○	○	○	○
16) Ich denke, den meisten MedizinstudentInnen ist digitale Medizin ein Begriff (mit den verschiedenen Aspekten, wie sie in diesem Modul vermittelt wurden).	○	○	○	○	○
17) Ich halte es für den richtigen Ansatz, eher Kompetenzen als rein theoretisches Wissen zu vermitteln.	○	○	○	○	○
18) Angehende MedizinerInnen sollten schon während des Studiums umfassend und frühzeitig gezielt zu Aspekten der digitalen Gesundheit geschult werden, z.B. durch Module wie dieses.	○	○	○	○	○
19) Lerninhalte digitaler Medizin sollten aus meiner Sicht auch im (Pflicht)Curriculum des Modellstudiengangs abgebildet werden.	○	○	○	○	○

20) Freie Kommentare zu diesem Unterthema (Digitale Medizin als Bestandteil des Medizinstudiums): ______________________________

Bitte teilen Sie uns noch kurz und prägnant ihre Gedanken zu folgenden Themen mit (gerne auch in Schlagworten):

21) In welcher Weise wird sich die Digitalisierung auf den Berufsalltag von Ärztinnen und Ärzten auswirken und wo sehen Sie die größten Herausforderungen? ____

22) Welche Inhalte und Kompetenzen der digitalen Medizin würden Sie empfehlen, an der Charité zu unterrichten? ____

23) Welche Lehrformate (z.B. Seminare, Workshops) halten Sie zur Lehre digitaler Medizin für am geeignetsten? ____

24) Optional können Sie hier noch Ihre Email-Adresse angeben, sollten Sie einer Kontaktaufnhame zu Ihrem Feedback zustimmen. __________________________________

Studierendenbefragung zum Wahlpflichtmodul „Digital Health“

(Kontakt: Daniel Glauert, Doktorand, daniel-leon.glauert@charite.de; PD Dr. Back, BwKrhs Berlin, DSFZ Charité, david.back@charite.de)

Sehr geehrte Studierende,

Bitte geben sie uns Rückmeldung zu Ihrer Erfahrung mit unserem gemeinsamen **Wahlpflichtmodul “Digital Health**”. Ihr Feedback wird uns bei der wissenschaftlichen Auswertung und Verbesserung des Lehrangebots helfen. Die Ergebnisse werden **anonymisiert** im Rahmen unseres Projekts „IDHEC“ (Implementation of a Digital Health Elective Class at a German Medical School) veröffentlicht.

Im Namen des gesamten Organisationsteams bedanken wir uns noch einmal sehr herzlich für Ihr Engagement und die gute Zusammenarbeit!

	**Organisation des Wahlpflichtmoduls**	stimme **voll** und ganz zu	Stim-me zu	neu-tral	Stim-me nicht zu	Stimme ganz und **gar nicht** zu
1.	Ich war mit der Organisation des Moduls zufrieden.	☐	☐	☐	☐	☐
2.	Ich war mit der Themenzusammenstellung des Moduls zufrieden.	☐	☐	☐	☐	☐
3.	Ich war mit dem Engagement der Dozierenden zufrieden.	☐	☐	☐	☐	☐
4.	Ich war mit der Kommunikation mit dem Organisationsteam zufrieden.	☐	☐	☐	☐	☐
5.	Ich bin mit meinem Wissenszuwachs durch das Wahlpflichtmodul zufrieden (soweit zutreffend: auch Fähigkeiten / Kompetenzen).	☐	☐	☐	☐	☐
6.	Ich würde das Wahlpflichtmodul meinen KommilitonInnen empfehlen.	☐	☐	☐	☐	☐
7.	Insgesamt bin ich mit dem Wahlpflichtmodul zufrieden.	☐	☐	☐	☐	☐

Freie Kommentarmöglichkeit:

____

____

	**Erfahrungen aus dem Wahlpflichtmodul**	stimme **voll** und ganz zu	Stim-me zu	neu-tral	Stim-me nicht zu	Stimme ganz und **gar nicht** zu
8.	Ich habe in diesem Rahmen erstmals im Studium Aspekte der digitalen Medizin gezielt vermittelt bekommen (wenn nein, bitte erläutern Sie dies im Kommentarfeld unten).	☐	☐	☐	☐	☐
9.	Ich fühle mich durch das Wahlpflichtmodul besser auf meinen zukünftigen Berufsalltag vorbereitet (v.a. auf potentielle digitale Aspekte).	☐	☐	☐	☐	☐
10.	Ich fühle mich durch das Modul bestärkt, mich in Zukunft weiter mit Aspekten digitaler Medizin zu beschäftigen	☐	☐	☐	☐	☐
11.	Ich würde noch mehr interdisziplinäre Lehrveranstaltungen in diesem Modul für sinnvoll halten (wenn ja, bitte erläutern Sie dies bitte konkret unten im Kommentarfeld).	☐	☐	☐	☐	☐
12.	Die gewählten Lehrformate haben das Lernen unterstützt.	☐	☐	☐	☐	☐
13.	Ich habe im Wahlpflichtmodul Aspekte meiner zukünftigen Arztrolle kennengelernt, die mir vorher nicht bewusst waren (Erfindergeist, Ethik, Recht, Ökonomie…)	☐	☐	☐	☐	☐

Freie Kommentarmöglichkeit:

____

____

Digitale Medizin im Medizinstudium	stimme **voll** und ganz zu	Stim-me zu	neu-tral	Stim-me nicht zu	Stimme ganz und **gar nicht** zu
14. Digitale Medizin wird den Berufsalltag von ÄrztInnen in den nächsten 5 Jahren stark beeinflussen.	☐	☐	☐	☐	☐
15. Digitale Medizin ist im aktuellen Medizinstudium aus meiner bisherigen Erfahrung ausreichend und gut abgebildet.	☐	☐	☐	☐	☐
16. Ich denke, den meisten meiner KommilitonInnen ist digitale Medizin ein Begriff (mit den verschiedenen Aspekten, wie sie in diesem Modul vermittelt wurden).	☐	☐	☐	☐	☐
17. Ich halte es für den richtigen Ansatz, eher Kompetenzen als rein theoretisches Wissen zu vermitteln.	☐	☐	☐	☐	☐
18. Angehende MedizinerInnen sollten schon während des Studiums frühzeitig und gezielt zu Aspekten der digitalen Gesundheit geschult werden, z.B. durch Module wie dieses.	☐	☐	☐	☐	☐
19. Lerninhalte digitaler Medizin sollten aus meiner Sicht auch im (Pflicht)Curriculum des Modellstudiengangs abgebildet werden.	☐	☐	☐	☐	☐

Freie Kommentarmöglichkeit:

____

____

Bitte teilen Sie uns noch kurz und prägnant ihre Gedanken zu folgenden Themen mit (gerne auch in Schlagwörtern):

20. In welcher Weise wird sich die Digitalisierung auf den Berufsalltag von ÄrztInnen auswirken und wo sehen Sie die größten Herausforderungen?

____

____

____

____

21. Das hat mir besonders an diesem Modul gefallen (Fächer, Inhalte, Abläufe etc.):

____

____

____

22. Das halte ich bei diesem Modul noch für verbesserungswürdig (Fächer, Inhalte, Abläufe etc.):

____

____

____

VIELEN DANK FÜR IHRE TEILNAHME!
