# Supplementary material for: Undergraduate Medical Competencies in Digital Health and Curricular Module Development: Mixed Methods Study
Source: J Med Internet Res. 2020 Oct 29;22(10):e22161. doi: 10.2196/22161 (PMC7661229; doi:10.2196/22161)
Supplement: Multimedia Appendix 3 [file jmir_v22i10e22161_app3.pdf]

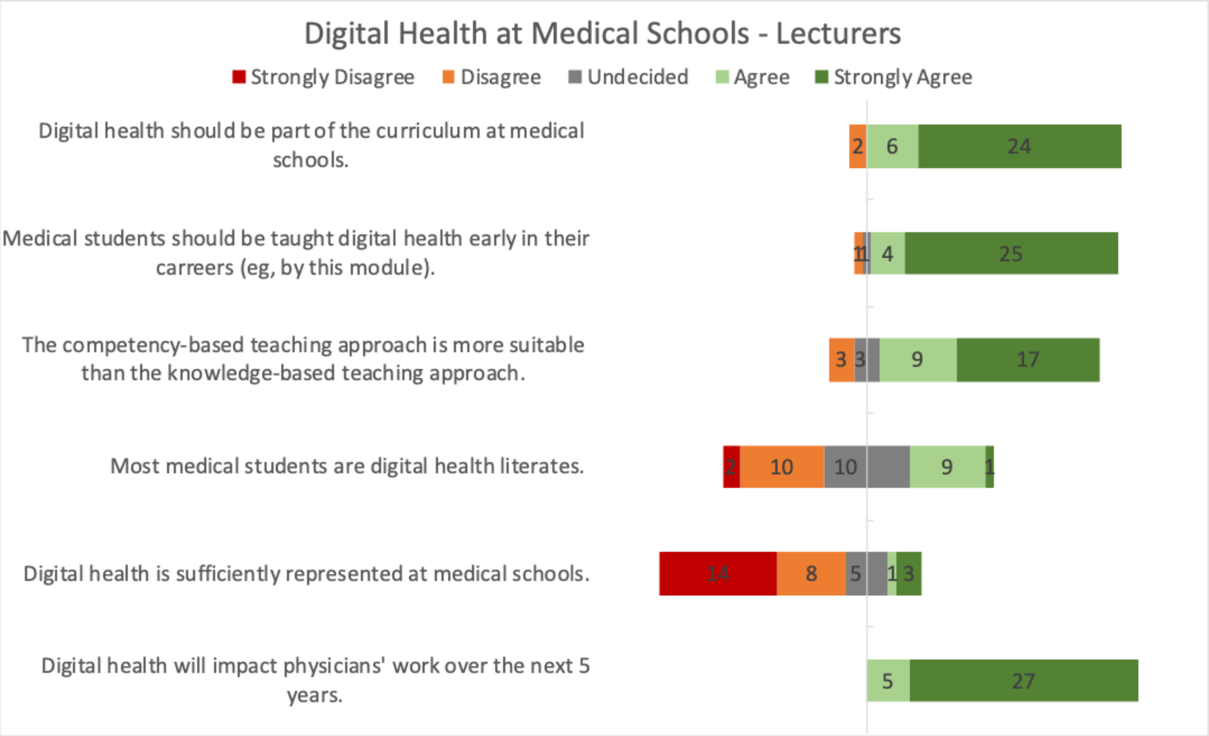
Digital Health at Medical Schools - Lecturers
Strongly Disagree
Disagree
Undecided
Agree
Strongly Agree
Digital health should be part of the curriculum at medical schools.
2
6
24
Medical students should be taught digital health early in their carreers (eg, by this module).
4
25
The competency-based teaching approach is more suitable than the knowledge-based teaching approach.
3
3
9
17
Most medical students are digital health literates.
10
10
9
1
Digital health is sufficiently represented at medical schools.
14
8
5
1
3
Digital health will impact physicians' work over the next 5 years.
5
27

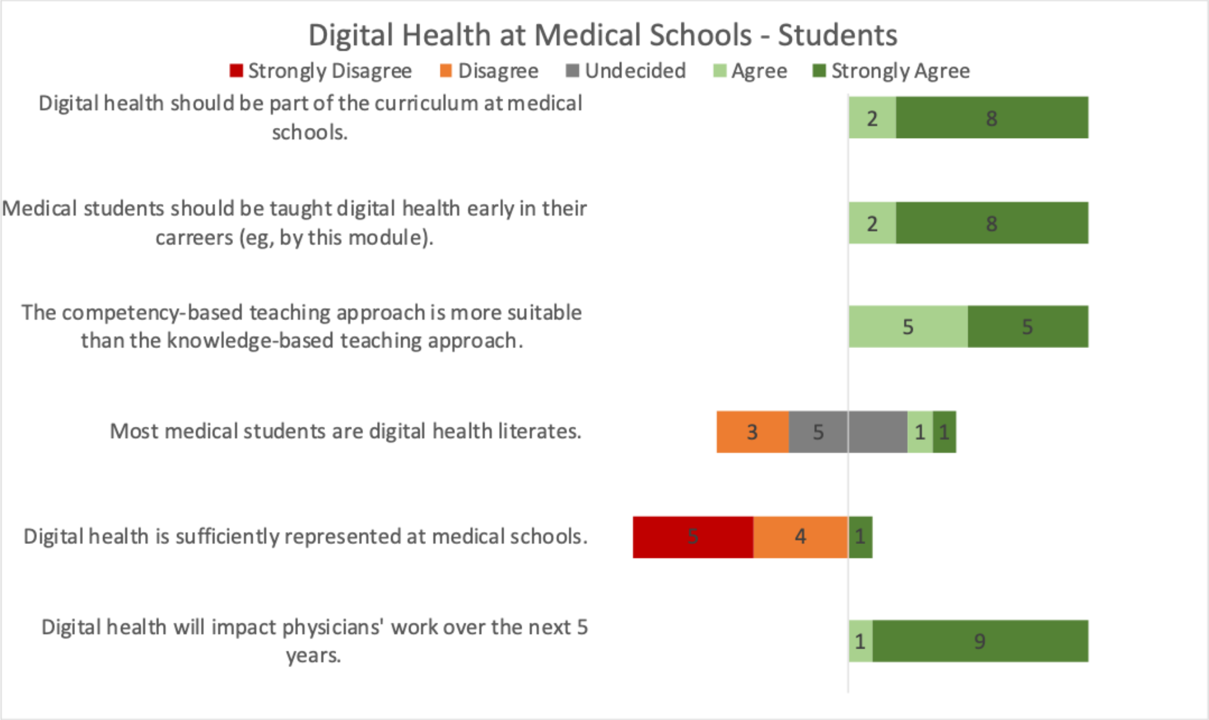
Digital Health at Medical Schools - Students
Strongly Disagree
Disagree
Undecided
Agree
Strongly Agree
Digital health should be part of the curriculum at medical schools.
2
8
Medical students should be taught digital health early in their carreers (eg, by this module).
2
8
The competency-based teaching approach is more suitable than the knowledge-based teaching approach.
5
5
Most medical students are digital health literates.
3
5
1
1
Digital health is sufficiently represented at medical schools.
5
4
1
Digital health will impact physicians' work over the next 5 years.
1
9

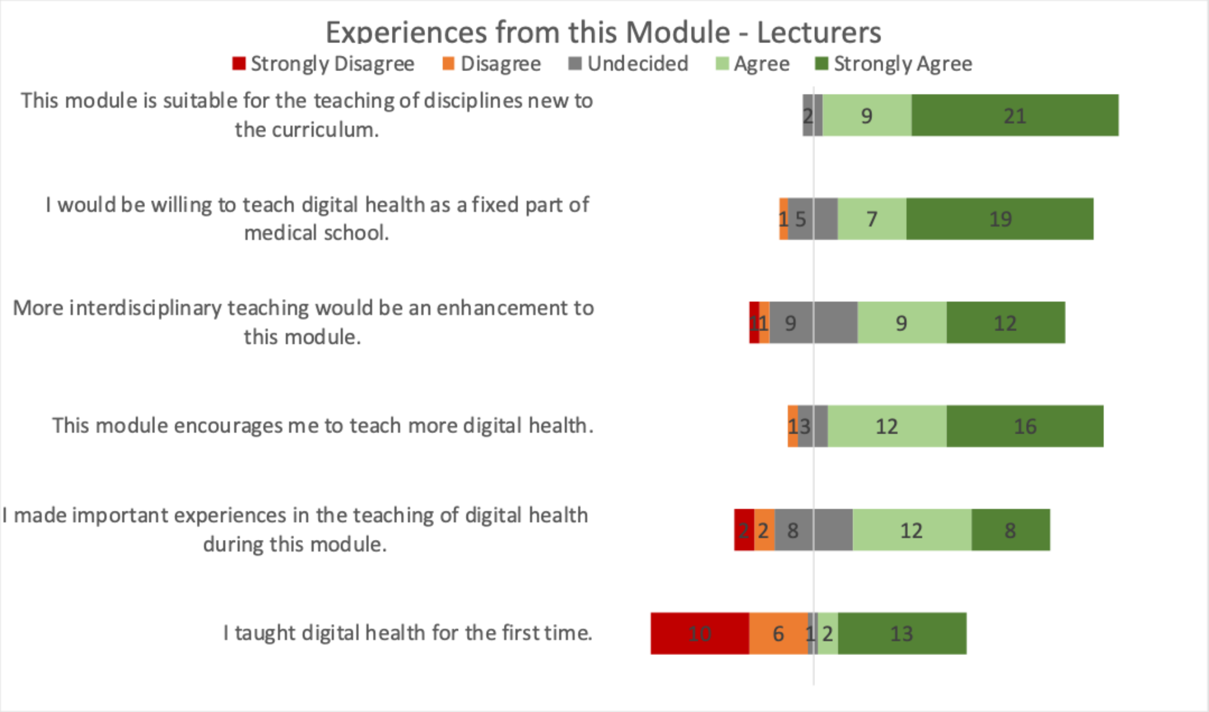
Experiences from this Module - Lecturers
Strongly Disagree
Disagree
Undecided
Agree
Strongly Agree
This module is suitable for the teaching of disciplines new to the curriculum.
2
9
21
I would be willing to teach digital health as a fixed part of medical school.
1
5
7
19
More interdisciplinary teaching would be an enhancement to this module.
1
9
9
12
This module encourages me to teach more digital health.
1
3
12
16
I made important experiences in the teaching of digital health during this module.
2
2
8
12
8
I taught digital health for the first time.
10
6
1
2
13

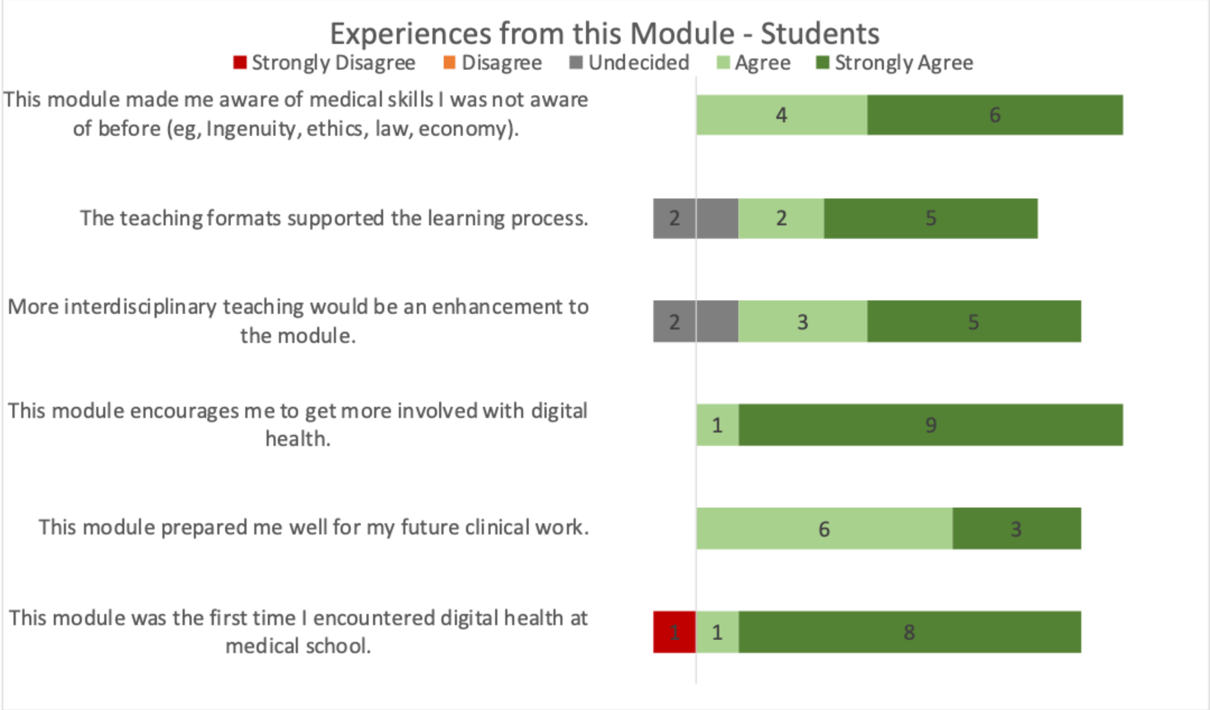
Experiences from this Module - Students
Strongly Disagree
Disagree
Undecided
Agree
Strongly Agree
This module made me aware of medical skills I was not aware of before (eg, Ingenuity, ethics, law, economy).
4
6
The teaching formats supported the learning process.
2
2
5
More interdisciplinary teaching would be an enhancement to the module.
2
3
5
This module encourages me to get more involved with digital health.
1
9
This module prepared me well for my future clinical work.
6
3
This module was the first time I encountered digital health at medical school.
1
1
8

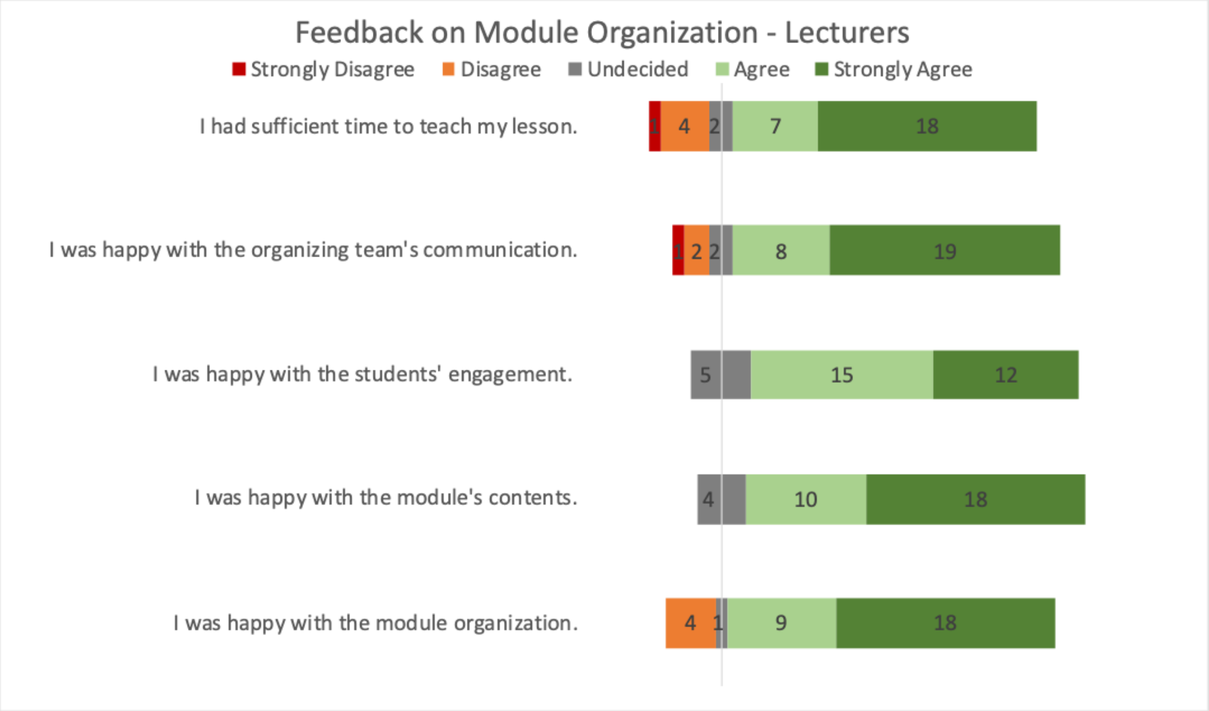
Feedback on Module Organization - Lecturers
Strongly Disagree
Disagree
Undecided
Agree
Strongly Agree
I had sufficient time to teach my lesson.
1
4
2
7
18
I was happy with the organizing team's communication.
1
2
2
8
19
I was happy with the students' engagement.
5
15
12
I was happy with the module's contents.
4
10
18
I was happy with the module organization.
4
1
9
18

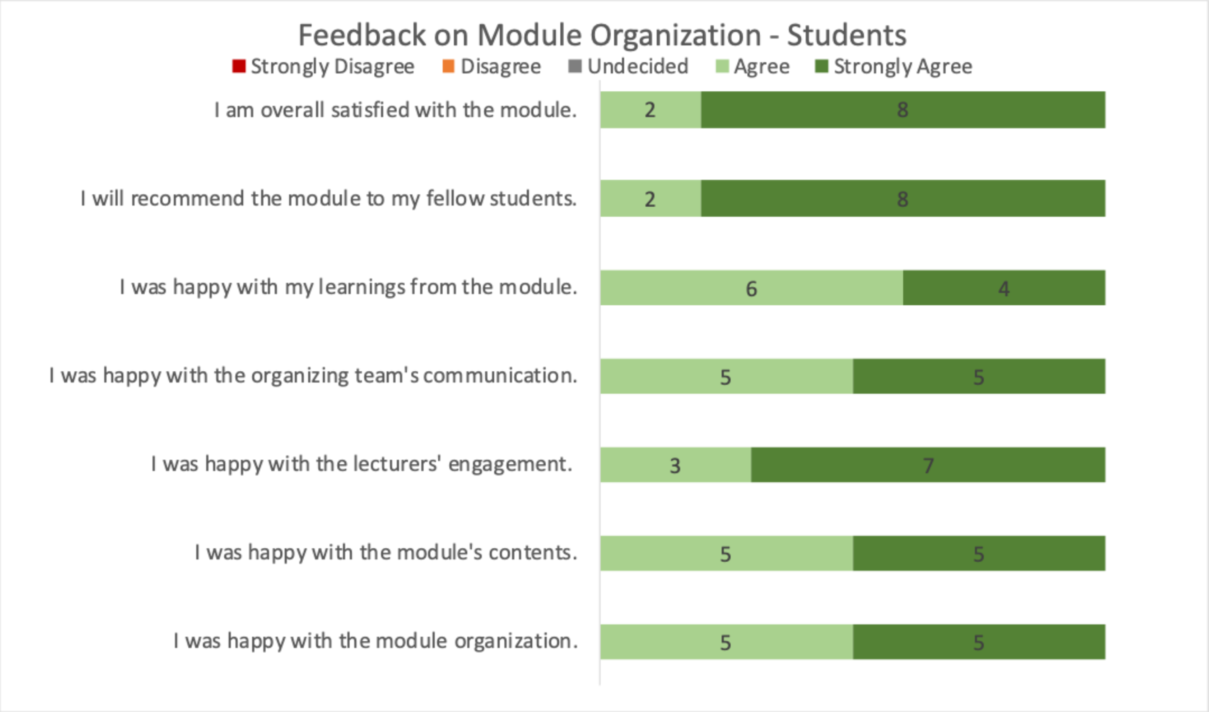
Feedback on Module Organization - Students
Strongly Disagree
Disagree
Undecided
Agree
Strongly Agree
I am overall satisfied with the module.
2
8
I will recommend the module to my fellow students.
2
8
I was happy with my learnings from the module.
6
4
I was happy with the organizing team's communication.
5
5
I was happy with the lecturers' engagement.
3
7
I was happy with the module's contents.
5
5
I was happy with the module organization.
5
5
